# Considerations for feature selection using gene pairs and applications in large-scale dataset integration, novel oncogene discovery, and interpretable cancer screening

**DOI:** 10.1186/s12920-020-00778-x

**Published:** 2020-10-22

**Authors:** Laura Moody, Hong Chen, Yuan-Xiang Pan

**Affiliations:** 1 Division of Nutritional Sciences, University of Illinois Urbana-Champaign, 461 Bevier Hall, 905 South Goodwin Avenue, Urbana, IL 61801 USA; 2 Department of Food Science and Human Nutrition, University of Illinois Urbana-Champaign, Urbana, IL USA; 3 Illinois Informatics Institute, University of Illinois Urbana-Champaign, Urbana, IL USA

**Keywords:** Gene pairs, Cancer, Top-scoring pair, Feature selection, Simplified tree ensemble learner

## Abstract

**Background:**

Advancements in transcriptomic profiling have led to the emergence of new challenges regarding data integration and interpretability. Variability between measurement platforms makes it difficult to compare between cohorts, and large numbers of gene features have encouraged the use black box methods that are not easily translated into biologically and clinically meaningful findings. We propose that gene rankings and algorithms that rely on relative expression within gene pairs can address such obstacles.

**Methods:**

We implemented an innovative process to evaluate the performance of five feature selection methods on simulated gene-pair data. Along with TSP, we consider other methods that retain more information in their score calculations, including the magnitude of gene expression change as well as within-class variation. Tree-based rule extraction was also applied to serum microRNA (miRNA) pairs in order to devise a noninvasive screening tool for pancreatic and ovarian cancer.

**Results:**

Gene pair data were simulated using different types of signal and noise. Pairs were filtered using feature selection approaches, including top-scoring pairs (TSP), absolute differences between gene ranks, and Fisher scores. Methods that retain more information, such as the magnitude of expression change and within-class variance, yielded higher classification accuracy using a random forest model. We then demonstrate two powerful applications of gene pairs by first performing large-scale integration of 52 breast cancer datasets consisting of 10,350 patients. Not only did we confirm known oncogenes, but we also propose novel tumorigenic genes, such as *BSDC1* and *U2AF1*, that could distinguish between tumor subtypes. Finally, circulating miRNA pairs were filtered and salient rules were extracted to build simplified tree ensemble learners (STELs) for four types of cancer. These accessible clinical frameworks detected pancreatic and ovarian cancer with 84.8 and 93.6% accuracy, respectively.

**Conclusion:**

Rank-based gene pair classification benefits from careful feature selection methods that preserve maximal information. Gene pairs enable dataset integration for greater statistical power and discovery of robust biomarkers as well as facilitate construction of user-friendly clinical screening tools.

## Background

Innovations in gene expression analysis have been critical in understanding the basic biological mechanisms underlying carcinogenesis as well as facilitating patient screening and stratification. For instance, transcriptomic profiling in breast and prostate cancer has been used to discover oncogenic gene fusions, identify at-risk patients, predict metastasis and recurrence, and determine appropriate treatment strategies for specific patient subgroups [1–7]. However, while genomic data can promote new discoveries and effective clinical decision-making, there are two major challenges regarding data integration and interpretability. First, constantly evolving technology makes it difficult to combine data across clinical trials and patient cohorts. This has ultimately led to separate studies with fewer patients, yielding a lack of reproducible, robust findings. Secondly, genome-wide analysis produces thousands of gene features, which can be challenging to parse and present in an interpretable manner. “Black box” techniques such as neural networks, support vector machines (SVM), and ensemble methods have proven successful in accurately classifying tumors and predicting patient prognosis, but such methods produce complex, nonlinear decision boundaries that are dependent on platform and data processing. Ultimately, additional effort is needed to translate findings into biologically meaningful outputs that can also be implemented in a clinical setting.

Utilizing gene pairs can enable rapid data integration and can be easily incorporated into an interpretable clinical framework. Gene pairs were first explored by Geman et al. and later by Tan et al. in the top-scoring pair (TSP) classifier [8]. The algorithm first makes within-sample pairwise comparisons between genes and then makes group comparisons by examining the number of relative reversals. Consider a set of *p* genes whose expression is measured in a transcriptomic profile *X* = {*x*_1_, *x*_2_, …, *x*_*p*_}. In this example, each X may belong to a particular class C = {1, 2}. For the gene pair (*x*_*i*_, *x*_*j*_), the calculation of the pair score proceeds by first observing the probability of *x*_*i*_ < *x*_*j*_ in class 1 [*p*_*ij*_ (*1*) = *P* (*x*_*i*_ < *x*_*j*_|*c = 1)]* and comparing it to the probability of x_*i*_ < x_j_ in class 2 [*p*_*ij*_ (*2*) = *P* (*x*_*i*_ < *x*_*j*_|*c = 2)]*. The difference between the two probabilities can then be calculated to get the pair score: TSP_*ij*_ = |*p*_*ij*_ (1) − *p*_*ij*_ (2)|.

The benefits of the TSP algorithm lie in its simplicity. The pair score is based on within-sample probabilities that do not rely on actual expression values. Thus, only within-sample gene ranks are necessary to perform the calculations, eliminating the need for standardized gene expression quantification techniques as well as data normalization methods. This is particularly well-suited for gene expression data. While laboratory values such as blood pressure and serum hemoglobin have standard units (e.g. mm Hg and g/dL), gene expression is routinely measured using several platforms and can be presented as microarray fluorescence intensity, sequencing reads, or PCR cycle number. Gene pairs are invariant to measurement platform or specific cutoffs. Additionally, TSP provides an interpretable alternative to “black box” methods. The algorithm simply identifies gene pairs whose expression is inverted between classes. Thus, there is no complex relationship between gene expression and class label. Overall, TSP is easy to implement and can be interpreted to produce biologically and clinically meaningful results.

Although several studies have used TSP to stratify patients and predict tumor progression, there are several methodological improvements and applications that have yet to be explored. One major limitation of TSP is information loss. The pair score only compares expression between two genes to find which is higher but does not take into account the magnitude of the difference. Furthermore, the use of probabilities does not account for the sample size of each class. It is desirable to retain more information while preserving the simplicity of the algorithm. This might be achieved through utilizing gene ranks rather than the number of relative reversals. Tan et al. applied this approach to break ties among pair scores, but incorporating ranks into the pair score itself could further improve reproducibility [9]. Wang et al. also devised a method to utilize sample size information in the calculation of the pair score, which improved classification accuracy on several cancer datasets [10]. Other beneficial modifications to the TSP classifier involve more powerful classification methods. While the TSP classifier employs a simple voting scheme, a handful of studies have paired TSP feature selection with more complex classification algorithms. For instance, feature selection via TSP has been used to train SVMs for patient stratification and prognostic prediction in cancer [11, 12]. By filtering noise and accounting for highly intricate data structures, the combination of TSP and SVM improved performance over each individual method alone.

**Fig. 1:**
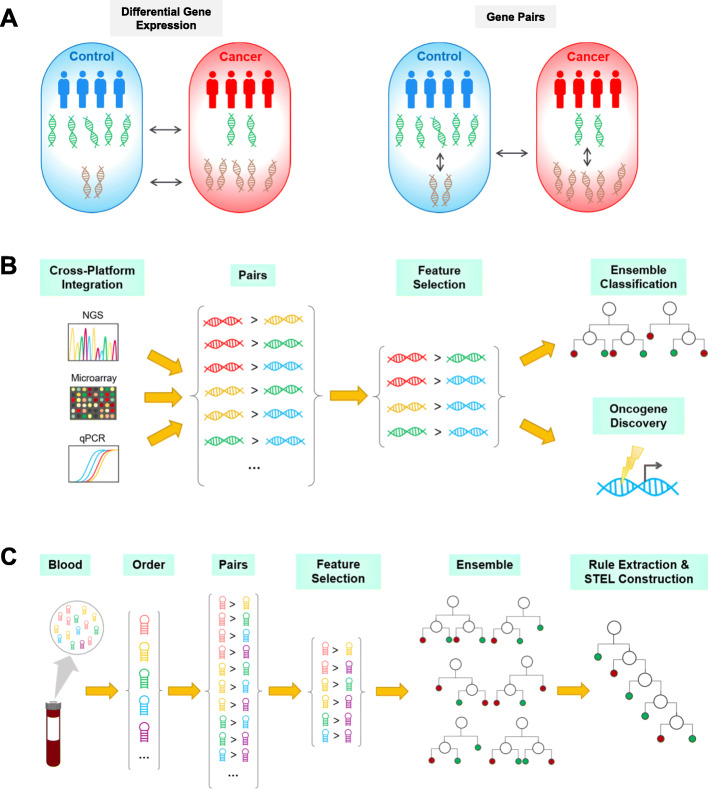
Strategies for using gene pairs to identify salient oncogenes and construct cancer screening tools. **a** Traditionally, differential gene expression is measured by comparing group means of individual genes. Gene pair analysis uses within-patient pairwise comparisons to obtain relative gene expression levels before making group comparisons. Thus, gene pairs only depend on gene rankings, not actual expression values. **b** This ranking-based methodology allows for integration of data across platforms. Next-generation sequencing (NGS), microarray, and qPCR all use different units for gene expression, but all three forms of data can be adapted to the gene pair framework. First, genes are ranked within each sample, enabling datasets to be combined. Next, pairwise comparisons are made in an exhaustive manner and feature selection is performed using filtering methods. The selected gene pairs can then be used for classification using ensemble methods. Also, the top features can be examined for their role in carcinogenesis. The use of gene pairs facilitates dataset integration in order to increase sample size and statistical power for robust oncogene detection. **c** Another application of gene pairs involves transparent and interpretable clinical screening tools. Circulating miRNA pairs can be used for cancer screening. First, miRNA in blood samples are quantified and ordered by expression level within each patient. Pairwise comparisons are made and feature selection is performed. Ensemble classifiers can then be built. In order to create more interpretable models, important rules are extracted and simplified tree ensemble learners (STEL) are constructed. This test is highly practical in a clinical setting because it is noninvasive, and the use of within-patient values is invariant to measurement platform and does not require the use of specific cutoffs or standard values

Here, we examine the efficacy of gene pairs in identifying robust cancer biomarkers and demonstrate how they might be implemented for clinical use (Fig. 1). We first evaluate the performance of five feature selection methods on simulated gene-pair data. Along with TSP, we consider other methods that retain more information in their score calculations, including the magnitude of gene expression change as well as within-class variation. We then show two clinical applications of gene pairs (Fig. 1). First, we validated known genetic biomarkers and proposed novel tumorigenic genes by performing feature selection after large-scale dataset integration. Using a ranking system, we were able to overcome heterogeneity in gene expression measurement technology and combine a total of 52 datasets containing over 10,000 breast tumors, which enabled us to identify robust tumor biomarkers. Finally, tree-based rule extraction was applied to serum microRNA (miRNA) pairs in order to devise a noninvasive screening tool for pancreatic and ovarian cancer.

## Results

### Comparison of feature selection methods on simulated data

While transcriptomic data contains thousands of gene features, the use of gene pairs dramatically increases the feature space. To avoid overfitting and minimize computational time, careful feature selection is critical. Data were simulated to test classification accuracy using several feature selection methods. Data structure was varied in terms of the effect size of differentially expressed genes, covariance structure, and number of genes with low expression. Each simulation contained 1000 genes, including 900 noise genes and 100 signal genes. The signal genes represented differentially expressed genes and followed multivariate normal distributions: *N*(*μ*, Σ) for class 1 and *N*(−*μ*, Σ) for class 2. Here, *μ* represents the effect size of differentially expressed genes. To simulate a moderate effect size, *μ* was set as a vector containing 10 unique values ranging from − 0.25 to 0.25 at increments of 0.05 (not including 0). Each value was repeated 10 times for a total of 100 values. A strong effect size was simulated by setting half of the values in *μ* to − 0.25 and the other half to 0.25.

Two covariance (Σ) structures were formulated in order to simulate oncogenes and tumor suppressor genes from certain pathways that were concurrently expressed. The covariance structure followed the general form:

$$ \Sigma =\left[\begin{array}{ccccc}1& \rho & \rho & \dots & \rho \\ {}\rho & 1& \rho & \dots & \rho \\ {}\vdots & \vdots & \ddots & \ddots & \vdots \\ {}\rho & \rho & \dots & \rho & 1\end{array}\right] $$

The signal genes were drawn from either an independent model with *ρ* = 0 or a correlated model with *ρ =* 0.6.

Finally, data were generated to mimic different types of noise. For the first scenario, all 900 noise genes were independently drawn from *N* (0, 1) distributions for both class 1 and class 2. For two other cases, we introduced genes that were expressed at very low levels with a few outliers. For these genes, 90% of the samples were drawn from the right side of a *N*(− 3, 0.1) distribution to mimic low or no expression, and 10% of the samples were drawn from a *N* (0, 1) distribution to represent technical error. Simulations were performed with 100 and 300 such genes, to simulate a dataset in which 10 and 30% of genes were expressed at low levels. For each simulation, a total of 400 samples were generated (200 per class). Half of the samples from each class were randomly chosen as the training set, leaving the other half as the test set. Within each sample, genes were ranked in descending order from 1 to 1000. Pairwise differences were calculated, generating new gene pair features with numeric values.

Five feature selection methods were used to identify top pairs. First, TSP score as defined by Geman et al. was employed. Absolute median (AM) and absolute average (AA) were computed by taking the absolute difference between the class median/average ranks. Also, Fisher median (FM) and Fisher average (FA) were calculated by first taking the weighted sum of the difference between each class median/average and the total median/average, then dividing by the weighted sum of the class variances. The methods were chosen because each includes a different amount of information in its score calculation. TSP is the most simplistic and only considers the probability of a difference in gene rank between classes. AA and AM are slightly more advanced and consider the magnitude of the difference. Finally, Fisher scores are the most complex and consider both the magnitude of the difference and the within-class coherence. Median was calculated as a counterpart to the mean in order to avoid outlier effects. TSP, AM, AA, FM, and FA scores were used to rank gene pairs such that higher values represent more important genes. All feature selection methods are described in detail in the Methods section.

After feature selection, the gene-pair feature values were then changed to a binary categorical variable representing either a negative or positive value. This was performed in order to reflect the type of data that might be observed in a clinical test. Random forest (RF) models were trained and prediction on the test set was performed. Model performance was evaluated using two metrics. First, classification accuracy on the test set was calculated using different numbers of gene pairs. Secondly, the percentage of selected features that contained at least one signal gene was measured. Each simulation was performed at least five times.

**Fig. 2:**
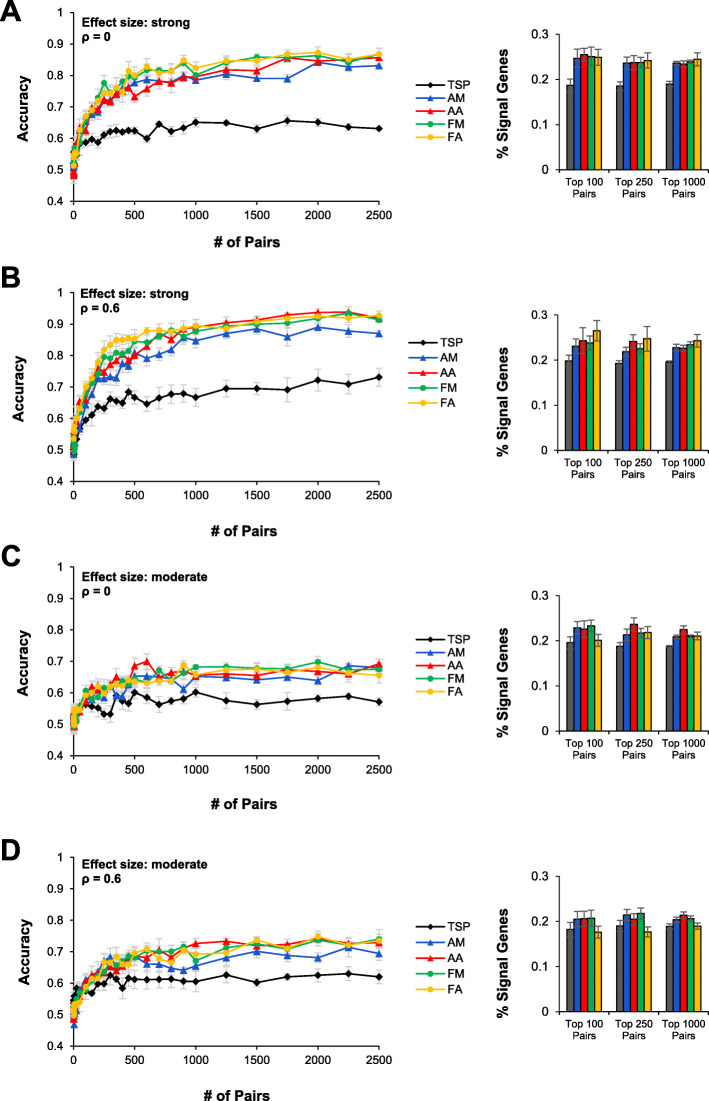
Performance of feature selection methods on simulated data with different effect sizes and covariance structures. Signal genes were simulated to follow one of four structures: **a** strong effect size and uncorrelated (*ρ* = 0), **b** strong effect size and correlated (*ρ* = 0.6), **c** moderate effect size and uncorrelated, and **d** moderate effect size and correlated. Performance of a random forest classifier was evaluated using classification accuracy on the test set as well as the percentage of identified gene pairs that contained at least one signal gene. All simulations were performed five times and data are presented as mean ± SEM

Model performance varied depending on effect size and covariance structure of the simulated data. As expected, for all feature selection methods, classification accuracy and identification of signal genes improved when the effect size was stronger (Fig. 2). The maximum accuracy for data with moderate versus strong effect size was 0.748 and 0.938, respectively. Moreover, when the effect size was strong, around 25% of gene pairs chosen by the feature selection methods (except TSP) contained at least one signal gene (Fig. 2). When the effect size was moderate, only around 20% of chosen features contained at least one signal gene. Similarly, accuracy for all feature selection techniques increased when signal genes were more correlated (*ρ* = 0.6). Under strong conditions, maximum accuracy increased from 0.868 in the independent data to 0.938 in the correlated data, and under moderate conditions, maximum accuracy increased from 0.692 in the independent data to 0.748 in the correlated data. In all cases, TSP performed the worst, while AM, AA, FM, and FA performed equally well (Fig. 2). TSP consistently yielded accuracies 10 and 20% lower than the other four methods on simulations with moderate and strong effect sizes, respectively.

**Fig. 3:**
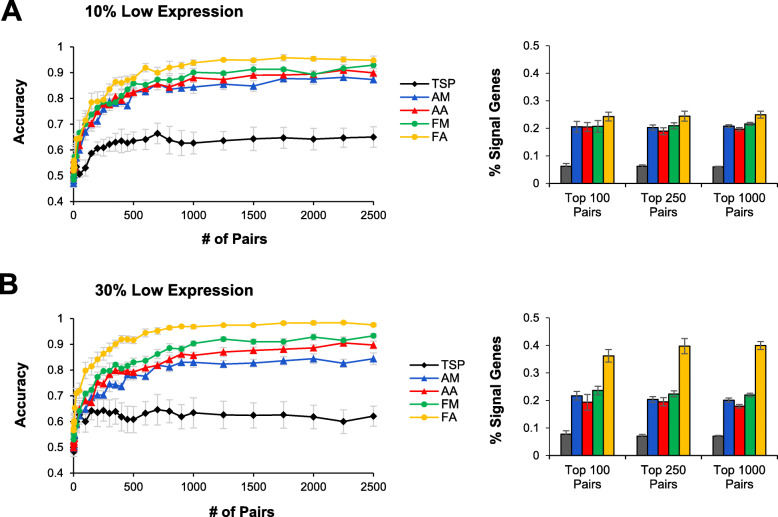
Performance of feature selection methods on simulated data with low gene expression. Noise was introduced to mimic genes with no or very low expression, representing (**a**) 10% or (**b**) 30% of the total features. Performance of a random forest classifier was evaluated using classification accuracy on the test set as well as the percentage of identified gene pairs that contained at least one signal gene. All simulations were performed five times and data are presented as mean ± SEM

When lowly-expressed genes were introduced as noise, FA performed significantly better than other selection methods (Fig. 3). Gene expression depends on several factors such as tissue type, developmental stage, environmental stimuli, and metabolic demands. Thus, certain genes are often completely turned off under particular conditions. These genes can be considered as noise and were simulated as 10% or 30% of total genes. For these simulation, other parameters were held constant such that the effect size was strong and the signal genes were correlated (ρ = 0.6). FA was particularly robust against genes with low expression, and was able to achieve maximum accuracy of over 0.95 and recovered significantly more signal genes than the other four methods in both simulations (Fig. 3). In terms of test accuracy, FM performed better than AA, and AA performed better than AM. This was particularly evident when there 30% of the genes had low expression. TSP was significantly worse than the other methods. FA also had higher accuracy and recovered more signal genes when there was 30% low expression compared to 10%. FM, AA, and TSP performed equally well on the 10 and 30% simulations. AM performed worse on the 30% simulation compared to the 10% simulation.

### Novel oncogene discovery in breast cancer

The use of gene pairs has many benefits over traditional gene expression values. Gene pairs remove the need for normalization and enable integration of datasets across platforms. Previous studies have been limited by sample size, but we attempted to overcome this limitation by combining 52 breast cancer gene expression datasets using gene rankings based on within-sample expression. This returned a total of 10,350 breast tumors and 1490 normal mammary tissue samples (Table 1). Next, the five feature selection methods were used to identify the top gene pairs between cancer and control. To validate the performance of the gene pairs, a RF classifier was built using the top 100 pairs. All five feature selection methods were able to distinguish tumor and normal tissue with at least 99% accuracy. Thus, it is correct to assume that the top pairs are likely relevant to cancer.

**Table 1:** Breast cancer datasets. Transcriptomic data from tumor and normal mammary tissue were downloaded from GEO. All datasets were combined to compare between cancer and control

Platform	Data Type	Control	Tumor	ER+	ER-	HER2+	HER2-	GSE ID
Affymetrix	Array	804	4193	1391	732	449	797	GSE4611, GSE7904, GSE10780, GSE10797, GSE11121, GSE15852, GSE18728^a^, GSE18864^a^, GSE20711^a^, GSE21653^a^, GSE22093^b^, GSE23988^b^, GSE26639^a^, GSE42568^b^, GSE45827, GSE48091, GSE48390^a^, GSE53031^a^, GSE54002, GSE65095^a^, GSE78958, GSE93601^b^, GSE124646, GSE129551^a^, GSE131027
Agilent	Array	618	1295	701	259	178	743	GSE21974^a^, GSE22820, GSE35186, GSE40206^a^, GSE43973, GSE49175, GSE49481^a^, GSE50939, GSE52604, GSE70905^a^, GSE70947^a^, GSE75678^a^, GSE80999^a^, GSE111601
Illumina	Array	0	1083	588	171	104	481	GSE20462, GSE36693, GSE37181^a^, GSE45725^a^, GSE46563^a^, GSE60785^a^, GSE103744^b^, GSE111563
Illumina	RNA-seq	68	3779	3186	337	524	3074	GSE47462, GSE81538^a^, GSE96058^a^, GSE99680^a^, GSE129508^a^
	**Total:**	**1490**	**10,350**	**5866**	**1499**	**1255**	**5095**	**52 datasets**

^a^Dataset used for classification based on ER and HER2 status. ^b^ Dataset used for classification based on ER status

**Fig. 4:**
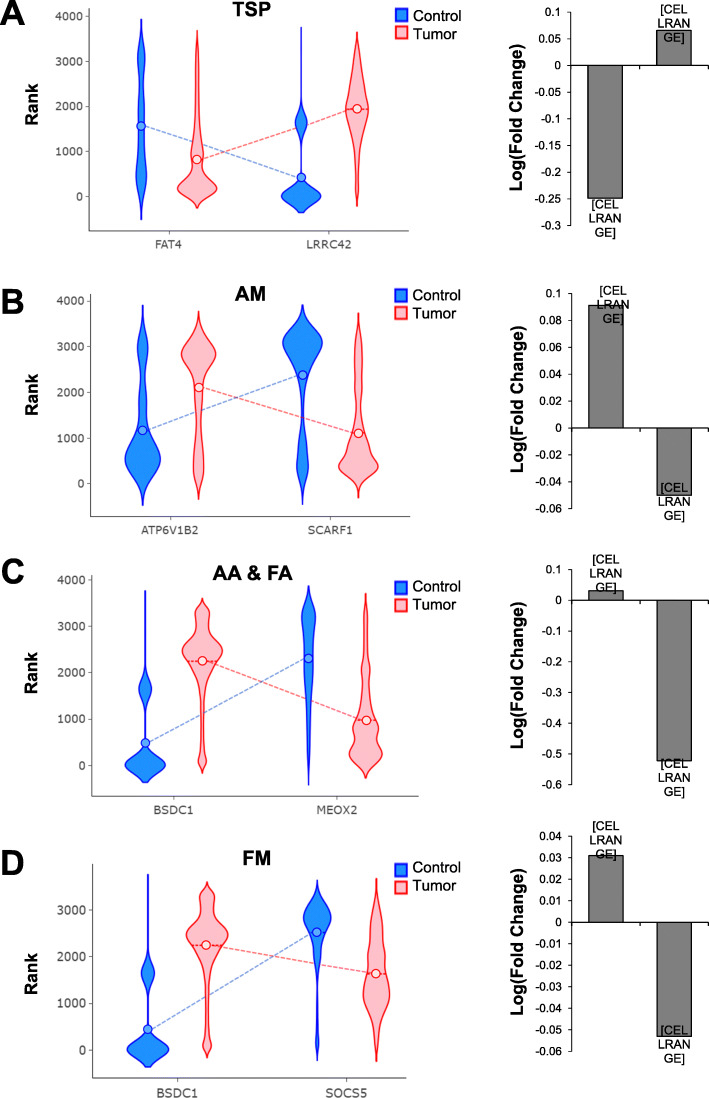
Top gene pairs identified by feature selection to distinguish between tumor and normal mammary tissue. Breast cancer gene pairs were filtered using feature selection. The highest scoring pair using **a** TSP, **b** AM, **c** AA and FA, and **d** FM are reported. Bar graphs depict the log (fold change) in gene expression in tumor compared to control tissue

We first examined the top pair from each method. TSP identified FAT atypical cadherin 4 (*FAT4*) and leucine rich repeat containing 42 (*LRRC42*), AM identified ATPase H+ transporting V1 subunit B2 (*ATP6V1B2*) and scavenger receptor class F1 (*SCARF1*), FM identified suppressor of cytokine signaling 5 (*SOCS5*) and BSD domain containing 1 (*BSDC1*), and both AA and FA identified *BSDC1* and mesenchyme homeobox 2 (*MEOX2*; Fig. 4). The top 10 pairs identified by each feature selection method are listed in Table 2 and the top 100 pairs are available in Supplementary file 1.

**Table 2:** Top ten gene pairs to distinguish between breast cancer and normal mammary tissue

Method	Gene 1	Gene 2	Score
TSP	FAT4	LRRC42	0.8391
	BTBD7	LRRC42	0.8381
	C11orf63	LRRC42	0.8344
	BSDC1	DENND3	0.8297
	BSDC1	BTBD7	0.8277
	CDR2L	FAT4	0.8266
	PLEKHA6	SPATA6	0.8241
	LRRC42	ZHX3	0.8239
	LRRC42	TEK	0.8228
	BSDC1	GRK5	0.8219
AM	ATP6V1B2	SCARF1	3948.5
	BSDC1	NRG2	3837.0
	PMPCA	SCARF1	3722.0
	ARHGEF5	BSDC1	3713.3
	ATP6V1H	NRG2	3708.5
	BSDC1	CSAD	3684.3
	ATP6V1B2	NRG2	3666.0
	FAF1	SCARF1	3642.3
	ERGIC2	SCARF1	3638.0
	BSDC1	SEMA3G	3613.8
AA	MEOX2	BSDC1	3127.9
	NRG2	BSDC1	3088.2
	SCARF1	BSDC1	3045.9
	MEOX1	BSDC1	3039.5
	GPRASP1	BSDC1	3019.9
	PPP1R1A	BSDC1	2980.3
	SEMA3G	BSDC1	2950.1
	CSAD	BSDC1	2930.1
	BSDC1	ARHGEF15	2926.7
	GNAZ	BSDC1	2882.6
FM	SOCS5	BSDC1	1.2573
	NRG2	BSDC1	1.0630
	SOCS5	LRRC42	1.0207
	NRG2	LRRC42	1.0186
	SEMA3G	BSDC1	1.0146
	RBM19	BSDC1	1.0085
	TBC1D22A	BSDC1	1.0067
	DYRK3	BSDC1	0.9893
	SEMA3G	LRRC42	0.9783
	NRG2	CDR2L	0.9721
	TBC1D22A	LRRC42	0.9663
FA	BSDC1	MEOX2	0.7516
	MEOX2	PLEKHA6	0.7231
	CDR2L	NRG2	0.6988
	LRRC42	MEOX2	0.6822
	BSDC1	SOCS5	0.6820
	BSDC1	NRG2	0.6729
	BSDC1	SEMA3G	0.6632
	LRRC42	NRG2	0.6557
	BSDC1	GJA4	0.6537
	NRG2	PLEKHA6	0.6530

Next, we compared our results to traditional group-comparison methods. Due to differences in data formats as well as methods and platforms used for gene expression measurement, it is not accurate to combine datasets for this analysis. Instead, we chose to evaluate one dataset (GSE93601) that contained the most samples from both tumor and control tissue. Fold change in gene expression in tumor versus control tissue was calculated for the top genes identified by gene-pair analysis (Fig. 4). False discovery rate *p*-values were used to determine significant differences in gene expression. As expected, *FAT4*, *SCARF1*, *MEOX2*, and *SOCS5* were significantly underexpressed in tumors (*p* = 1.48e-52, *p* = 6.31e-4, *p* = 4.43e-54, and *p* = 9.09e-4, respectively). Conversely, *LRRC42*, *ATP6V1B2*, and *BSDC1* were significantly overexpressed (*p* = 1.79e-9, *p* = 8.35e-9, and *p* = 0.025, respectively).

**Fig. 5:**
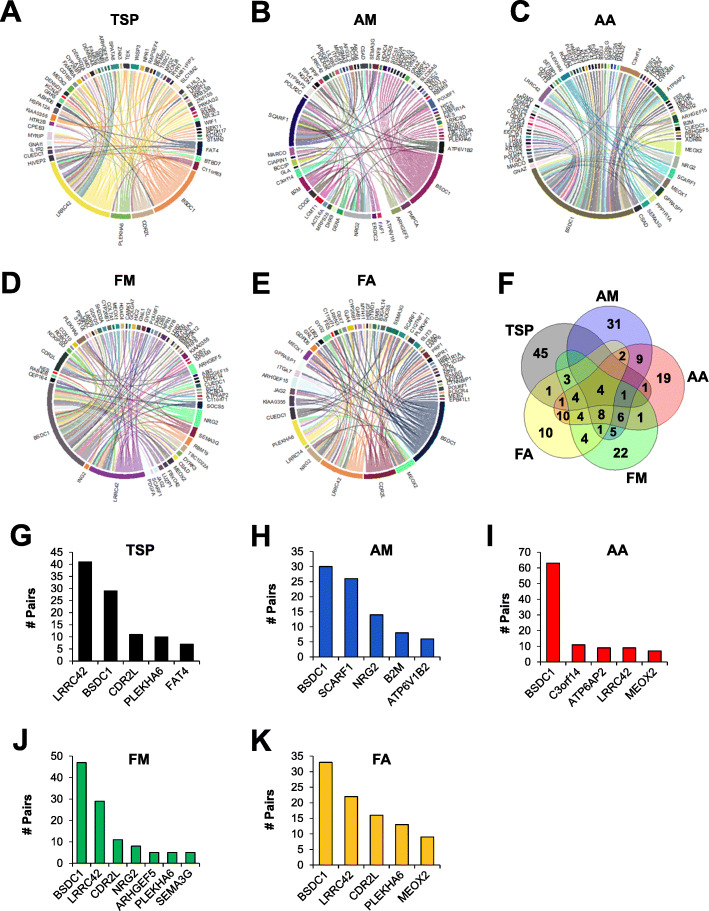
Gene pairs facilitate large-scale dataset integration to identify BSDC1 and LRRC42 as novel breast cancer biomarkers. Transcriptomic profiles from 10,350 breast tumors were combined and the top 100 gene pairs were filtered using **a** TSP, **b** AM, **c** AA, **d** FM, and **e** FA. Connected genes represent a pair. **f** Genes within the top 100 pairs were often common to multiple feature selection methods. The top five most represented genes using **g** TSP, **h** AM, **i** AA, **j** FM, and **k** FA are reported. Y-axes represent the number of times the gene occurs in the top 100 pairs

We then visualized the genes contained within the top 100 pairs using each method (Fig. 5). Of the top 100 pairs, TSP pairs contained 60 unique genes, AM pairs contained 67 genes, AA pairs contained 70 genes, FM pairs contained 63 genes, and FA pairs contained 52 genes. In terms of genes included in the top 100 pairs, there was considerable overlap between feature selection methods. All five methods identified *BSDC1*, *LRRC42*, *MEOX2*, and plekstrin homology domain containing A6 (*PLEKHA6*) as a gene in the top 100 pairs. Four out of five methods found gene pairs containing rho guanine nucleotide exchange factor 15 (*ARHGEF15*), cysteine sulfinic acid decarboxylase (*CSAD*), mesenchyme homeobox 1 (*MEOX1*), neuregulin 2 (*NRG2*), POU class 6 homeobox 1 (*POU6F1*), *SCARF1*, semaphoring 3G (*SEMA3G*), and *SOCS5* (AM, AA, FM, and FA), or cerebellar degeneration related protein 2 like (*CDR2L*), CUE domain containing C1 (*CUEDC1*), cytochrome P450 family 26 subfamily B1 (*CYP26B1*), and dystrophin (*DMD*) (TSP, AA, FM, FA), or nuclear receptor subfamily 3 group C2 (*NR3C2*) (TSP, AM, AA, FM). Overall, TSP appeared to be least similar to the other four methods, as 75% of the identified genes were unique. The other four methods shared 42.9% of their genes, and concordance was particularly high between AA and FA, which shared 37.1% of their genes.

We also examined the top five most represented genes in each feature selection method. *BSDC1* was among the top five in all feature selection methods. *LRRC42* was observed in four of the methods, *CDR2L* and *PLEKHA6* were observed in three methods, and *NRG2* and *MEOX2* were observed in two methods. Finally, we report genes that were represented in at least 10% of the top 100 genes in at least one feature selection method: *BSDC1*, chromosome 3 open reading frame 14 (*C3orf14*), *CDR2L*, *LRRC42*, *NRG2*, *PLEKHA6*, and *SCARF1* (Table 3). Interestingly, *C3orf14*, *CDR2L*, *LRRC42*, *MEOX2*, *NRG2*, and *SCARF1* are known carcinogenic genes, while *BSDC1* and *PLEKHA6* have not been described in previous cancer literature. Overall, we demonstrate the ability of gene pairs to combine datasets and validate the performance of feature selection methods by identifying known tumorigenic genes and other potential oncogenes.

**Table 3:** Gene pair identification of novel tumorigenic genes to distinguish between breast cancer and control tissue. Genes represent those that were present in at least 10% of the top 100 pairs in at least one of the feature selection methods. Feature selection columns denote the number of occurrences in the top 100 gene pairs

Gene Symbol	Gene Name	TSP	AM	AA	FM	FA	Function	Location
BSDC1	BSD domain containing 1	29	30	63	47	33	Unknown	1p35.1
C3orf14	Chromosome 3 open reading frame 14	0	4	11	0	0	Unknown	3p14.2
CDR2L	Cerebral degeneration related protein 2 like	11	0	1	11	16	Antigen for onconeural antibody (Yo)	17q25.1
LRRC42	Leucine rich repeat containing 42	41	3	9	29	22	Nuclear protein	1p.32.3
NRG2	Neuregulin 2	0	14	6	8	8	Growth factor	5q31.2
PLEKHA6	Pleckstrin homology domain containing A6	10	1	5	5	13	Intracellular signaling	1q32.1
SCARF1	Scavenger receptor class F1	0	26	5	1	3	LDL receptor	17p13.3

### Classification of breast tumor subtypes

**Fig. 6:**
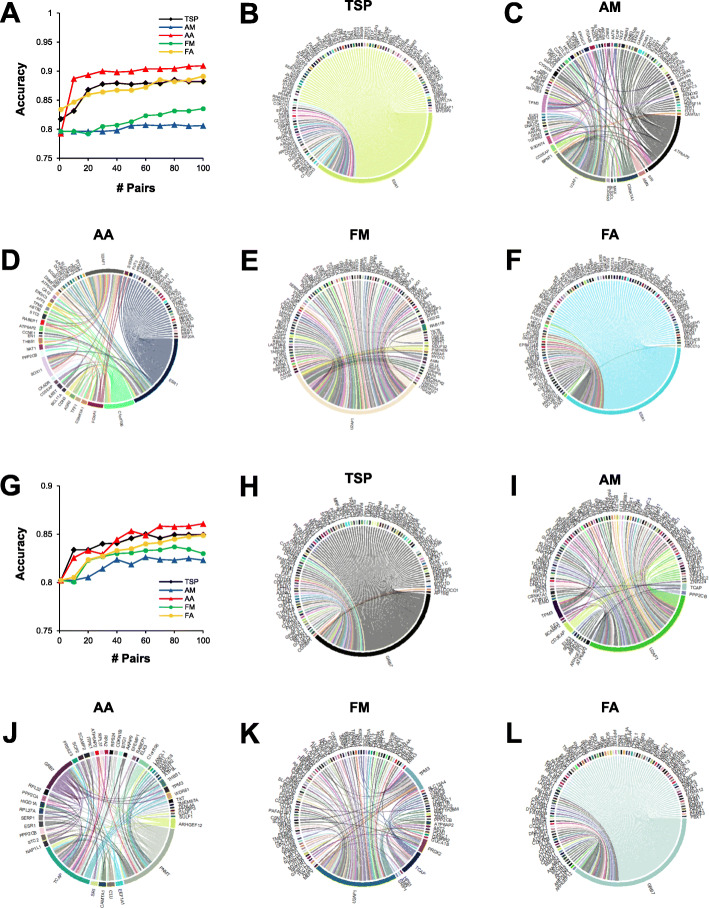
Gene pairs enable tumor stratification based on ER and HER2 status. **a** Patients were stratified based on ER status. Gene pair features were filtered and a random forest classifier was built to measure classification accuracy. The top 100 gene pairs were identified using **b** TSP, **c** AM, **d** AA, **e** FM, and **f** FA. **g** Patients were then divided by HER2 status. Gene pair features were filtered and a random forest classifier was built to measure classification accuracy. The top 100 gene pairs were identified using **h** TSP, **i** AM, **j** AA, **k** FM, and **l** FA. Connected genes represent a pair

After showing that gene pairs could identify important known and novel oncogenes, we then focused on the ability of gene pairs to classify breast cancer subtypes. There were 29 datasets that contained information regarding patients’ estrogen receptor (ER) status (Table 1). A total of 5866 ER-positive and 1499 ER-negative patients were surveyed. Genes were ranked within each sample, and samples were divided into training (75%) and testing (25%) sets. Feature selection was performed using the five methods, and a RF classifier was constructed. Classification accuracy on the testing data was evaluated using the top 100 pairs. Feature selection on gene pairs was successful, as top gene pairs from each method could classify patients with a maximum accuracy of least 0.80 (Fig. 6). AA pairs returned the best accuracy (0.909), while AM performed the worst (0.808). The top 100 gene pairs identified with each feature selection method are listed in Supplementary file 2.

We expected that ER expression would be very different between ER-positive and ER-negative patients, so it is not surprising that TSP, AA, and FA each identified estrogen receptor 1 (*ESR1*) as the most represented gene in the top 100 pairs. Interestingly, *ESR1* was not found in any of the top 100 AM or FM pairs. Rather, U2 small nuclear RNA auxiliary factor 1 (*U2AF1*) was the most represented gene in top FM pairs, while both *U2AF1* and ATPase H+ transporting accessory protein 2 (*ATP6AP2*) were present in more than 30% of the top 100 AM pairs. Also, seven genes were present in at least 10% of the top 100 pairs in at least one of the feature selection methods: *ATP6AP2*, casein kinase 1 alpha 1 (*CSNK1A1*), *ESR1*, innate immunity activator (*INAVA*), SRY-box 11 (*SOX11*), tropomyosin 3 (*TPM3*), and *U2AF1* (Table 4). We validated that feature selection with TSP, AA, and FA could be used to identify *ESR1* as the primary gene by which to stratify ER-positive and ER-negative tumors; however, our findings using AM and FM also suggest that other highly represented genes may play a role in determining ER status.

**Table 4:** Top genes for classification of tumors based on ER status. Genes represent those that were present in at least 10% of the top 100 pairs in at least one of the feature selection methods. Feature selection columns denote the number of occurrences in the top 100 gene pairs

Gene Symbol	Gene Name	TSP	AM	AA	FM	FA	Function	Location
ATP6AP2	ATPase H+ transporting accessory protein 2	0	39	3	1	0	Vacuolar-ATPase, pH control	Xp11.4
CSNK1A1	Casein kinase 1 alpha 1	0	13	3	0	0	Tumor suppressor, Wnt/β-catenin signaling	5q32
ESR1	Estrogen receptor 1	100	0	45	0	99	Estrogen signaling	6q25.1–2
INAVA	Innate immunity activator	1	1	17	0	1	Adherens junctions	1q32.1
SOX11	SRY-box 11	0	0	12	0	2	Transcription factor, cell fate	2p25.2
TPM3	Tropomyosin 3	0	11	2	0	0	Stabilization of actin filaments	1q21.3
U2AF1	U2 small nuclear RNA auxiliary factor 1	1	34	22	93	1	Splicing	21q22.3

Patients were also stratified by HER2 status. For this analysis, 24 datasets with 1255 HER2-positive and 5095 HER2-negative samples were used. The five feature selection methods were used to find the top 100 pairs, and RF was performed to classify samples. The feature selection methods performed similarly and all achieved a maximum accuracy above 0.83. However, the best accuracy was found using AA pairs (0.861), while AM performed the worst (0.826; Fig. 6). We then visualized the top 100 pairs using each method (Fig. 6). Within the top 100 pairs, AM pairs contained 98 unique genes, AA pairs contained 47 genes, and FM pairs contained 103 genes. TSP and FA pairs contained 101 unique genes each and growth factor receptor bound protein 7 (*GRB7*) was present in every pair. The majority of the top 100 AM and FM pairs contained *U2AF1* (80 and 57%, respectively). Feature selection using AA was similar to TSP and FA in that 20% of the top pairs contained *GRB7*, but it was also unique in choosing 42 pairs with phenylethanolamine N-methyltransferase (*PNMT*) and 31 pairs containing titin-cap (*TCAP*). The top 100 gene pairs identified with each feature selection method are listed in Supplementary file 3.

Surprisingly, HER2 was not observed among the top 100 pairs using any of the feature selection methods. Instead, we identified five genes that were present in at least 10% of the top 100 pairs in at least one of the feature selection methods: *GRB7*, *PNMT*, *TCAP*, *TPM3*, and *U2AF1* (Table 5). *GRB7*, *PNMT*, and *TCAP* are expected to be amplified in HER2-positive breast cancers due to their location within the HER2 amplicon on the long arm of chromosome 17 (17q12) [13]. *TPM3* and *U2AF1* are perhaps novel biomarkers of HER2 status. It is unclear why *HER2* was not identified as a top gene, however it may be the case that the top genes possess characteristics that are more conducive to discovery by gene-pair techniques, such as lower within-group variation, greater between-group differences, or a gene partner that displays inverse expression.

**Table 5:** Top genes for classification of tumors based on HER2 status. Genes represent those that were present in at least 10% of the top 100 pairs in at least one of the feature selection methods. Feature selection columns denote the number of occurrences in the top 100 gene pairs

Gene Symbol	Gene Name	TSP	AM	AA	FM	FA	Function	Location
GRB7	Growth factor receptor bound protein 7	100	0	20	0	100	HER2 signaling pathway	17q12
PNMT	Phenolethanolamine N-methyltransferase	0	0	42	0	0	Catecholamine biosynthesis	17q12
TCAP	Titin-cap	0	4	31	16	0	Scaffolding protein	17q12
TPM3	Tropomyosin 3	0	12	3	1	0	Stabilization of actin filaments	1q21.3
U2AF1	U2 small nuclear RNA auxiliary factor 1	0	80	1	57	0	Splicing	21q22.3

### Blood-based cancer screening using miRNA pair trees

Another powerful application of gene pairs is cancer screening. Not only do pairs remove methodology constraints as well as the need for designating cutoffs, but when combined with a simplified prediction model, they may also provide the physician and patient with a transparent, highly interpretable screening procedure. For this analysis, we utilized circulating miRNA data from four cancer types. Blood miRNA are ideal biomarkers because they are more stable than mRNA and are collected in a noninvasive manner.

Serum miRNA data were downloaded for bladder, ovarian, pancreatic/biliary tract, and prostate cancer patients (Table 6). Gender-matched control serum samples were included for each cancer type. Data were randomly divided into training (75%) and testing sets (25%), and the five feature selection methods were used to find the top 100 miRNA pairs. RF and boosted tree (BT) models were trained using the top pairs, and the best model for each cancer type was chosen based on test accuracy (Table 7). For ovarian and pancreatic cancer, FM and FA produced higher accuracy than other methods. Prostate cancer was best classified with an accuracy of 0.780 using a RF with 80 AM features. None of the feature selection methods performed particularly well for bladder cancer classification, as accuracy remained below 0.65; however, a RF using 83 TSP features yielded an accuracy of 0.643 and outperformed other methods.

**Table 6:** Circulating miRNA datasets

Cancer Type	GSE ID	Control Train	Cancer Train	Control Test	Cancer Test
Bladder	GSE113486	294	294	98	98
Ovary	GSE106817	2069	240	690	80
Pancreas	GSE59856, GSE106817	516	235	172	78
Prostate	GSE112264	211	607	71	202

**Table 7:** Performance of feature selection methods and ensemble classifiers used to build STELs from circulating miRNA pairs

Cancer	Feature Selection	Model	Accuracy	Pairs	STEL Accuracy
Bladder	TSP	RF	0.643	83	0.622
	AM	RF	0.622	97	0.556
	AA	RF	0.628	91	0.577
	FM	RF	0.628	87	0.566
	FA	RF	0.638	94	0.577
	TSP	BT	0.633	99	0.602
	AM	BT	0.612	82	0.536
	AA	BT	0.622	96	0.531
	FM	BT	0.617	92	0.536
	FA	BT	0.617	97	0.541
Ovary	TSP	RF	0.901	90	0.896
	AM	RF	0.909	82	0.896
	AA	RF	0.905	59	0.896
	FM	RF	0.958	77	0.936
	FA	RF	0.956	87	0.927
	TSP	BT	0.899	96	0.896
	AM	BT	0.906	96	0.896
	AA	BT	0.904	78	0.896
	FM	BT	0.957	66	0.896
	FA	BT	0.960	98	0.896
Pancreas	TSP	RF	0.832	93	0.772
	AM	RF	0.712	81	0.688
	AA	RF	0.696	23	0.688
	FM	RF	0.868	69	0.848
	FA	RF	0.864	80	0.828
	TSP	BT	0.796	93	0.744
	AM	BT	0.708	39	0.688
	AA	BT	0.700	97	0.688
	FM	BT	0.900	97	0.772
	FA	BT	0.872	96	0.748
Prostate	TSP	RF	0.762	83	0.740
	AM	RF	0.780	80	0.766
	AA	RF	0.755	100	0.740
	FM	RF	0.747	8	0.740
	FA	RF	0.747	8	0.740
	TSP	BT	0.762	94	0.740
	AM	BT	0.762	36	0.740
	AA	BT	0.740	1	0.740
	FM	BT	0.740	1	0.740
	FA	BT	0.740	1	0.740

While we show that pairs can be used to classify certain tumors with high accuracy using ensemble methods, we wanted to examine whether simpler classification schemes would also produce favorable results. By using a transparent classification model, we hope to provide physicians with a user-friendly tool that could easily be implemented in the clinic. Thus, we performed rule extraction on RF and BT models in order to build simplified tree ensemble learners (STEL). As expected, there was some loss of accuracy in using STELs versus full ensemble models (Table 7). For ovarian cancer, the highest classification accuracy using a full BT model was 0.960 and was observed using 98 FA pairs. The highest STEL accuracy was 2.4% lower at 0.936. Pancreatic cancer classified with an accuracy of 0.900 using 97 FM pairs in a BT model, whereas the highest STEL accuracy was 5.2% lower at 0.848. For prostate cancer, RF-STEL using AM features yielded the highest accuracy in prostate cancer (0.766), which was 1.4% lower than the most accurate RF classifier. In bladder cancer, the RF-STEL gave the highest classification accuracy (0.622), which was 2.1% lower than the best RF classifier.

**Fig. 7:**
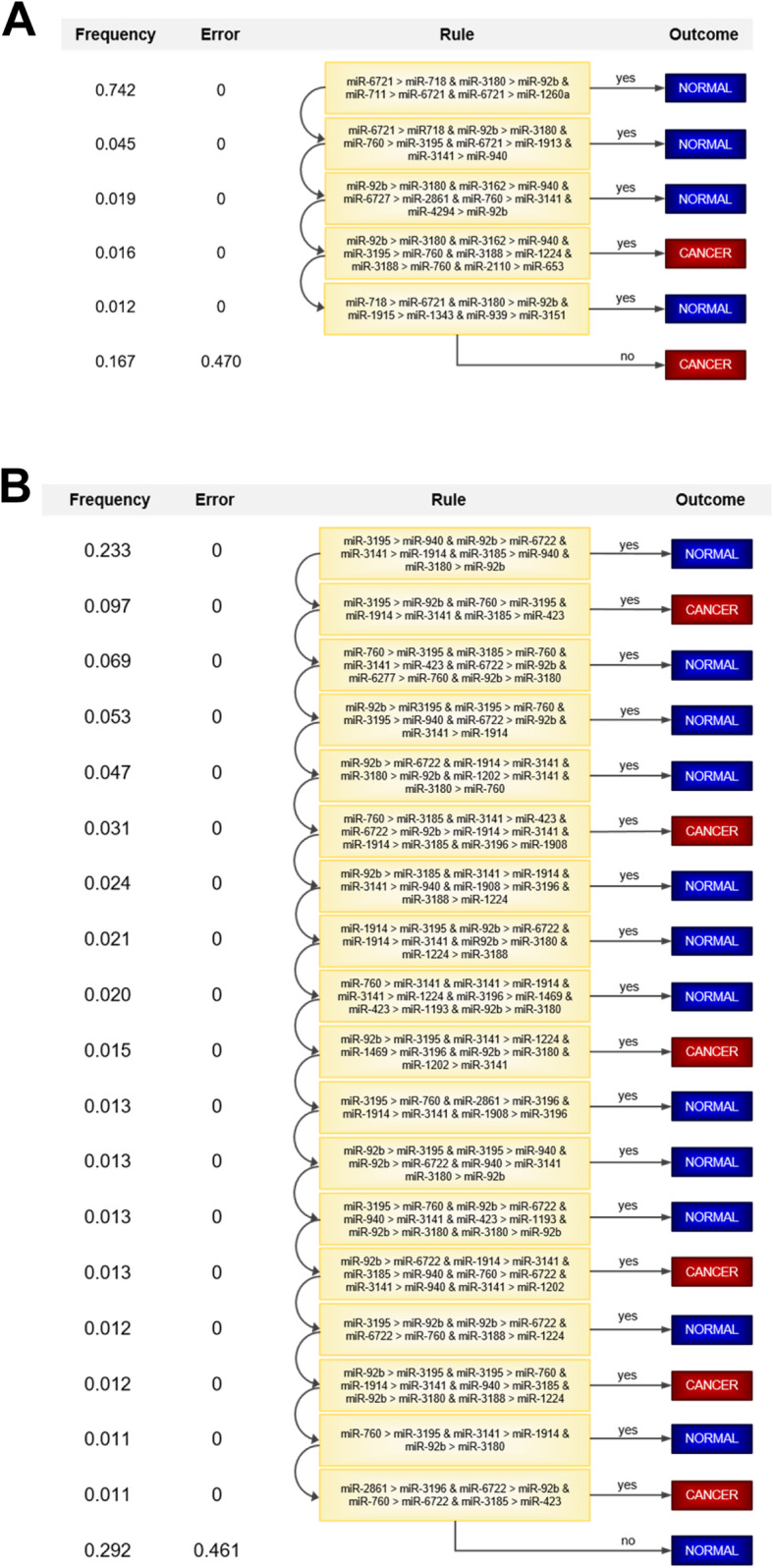
Simplified tree ensemble learners for ovarian and pancreatic cancer. Circulating miRNA were used to build STELs for (**a**) ovarian and (**b**) pancreatic cancer screening. Frequency refers to the frequency of cases in the dataset that satisfy the corresponding rule. Error refers to the error rate of the rule when the corresponding outcome is chosen

To demonstrate the interpretable nature of STEL models, we report the top-performing STEL classifiers for ovarian and pancreatic cancer. These simplified schemes provide a natural workflow that can be easily understood by physicians and patients. Furthermore, they reduce the number of miRNA in the model, which can expedite sample processing. Indeed, the STEL for ovarian cancer consisted of five rules, 17 miRNA pairs, and 23 unique miRNA (Fig. 7). The STEL for pancreatic cancer was more complex and included 18 rules and 25 miRNA pairs, but included only 18 unique miRNA. Overall, we provide an accessible framework by which miRNA pairs can be used to identify patients with tumors.

## Discussion

In the current study, we utilize rank-based methodology and gene pairs to integrate genomic datasets, identify novel oncogenes, and devise interpretable classifiers for cancer screening. First, we tested feature selection methods on simulated gene-pair data. Performance was dependent on covariance structure and the presence of genes with low expression; however, methods that preserved more information were generally better able to identify signal genes and produce higher accuracy using a random forest classifier. Next, we demonstrated the utility of gene pairs on real cancer data. We combined 52 datasets with over 10,000 breast tumors in order to discover novel oncogenes and robust biomarkers of tumor subtype. To our knowledge, we are the first to integrate gene expression datasets on such a large scale. We then capitalized on the simplicity of gene pairs and used circulating miRNA to create simplified tree ensemble learners for ovarian and pancreatic cancer screening.

We first conducted simulations to test five feature selection methods. Limiting the number of genes needed for classification not only reduces the risk of overfitting and improves model performance, but it could also lower clinical costs and streamline screening and diagnosis. Thus, feature selection is optimal. Although wrapper and embedded feature selection methods can account for feature dependencies and typically produce better classification results, they are computationally expensive because they interact with the classifier [14]. This is particularly important in our case because of the extremely large number of features. The use of gene pairs increases the number of features from *n* to $\frac{n\left(n-1\right)}{2}$. In our case, the combined breast cancer dataset contained a total of 3435 genes, which amounted to 5,897,895 pairs. We therefore opted to use filter methods to reduce computational cost. Filter methods are typically univariate in that they assume features are independent, but by considering gene pairs, we capture pairwise feature interactions while maintaining computational efficiency.

In our simulations, we found that feature selection methods that preserve more information typically yield better classification accuracy. We tested five feature selection methods that incorporated different amounts of information in their score calculation. TSP only considers whether or not there is a difference in gene rank between classes, AA and AM consider the magnitude of the difference, and Fisher scores consider the magnitude of the difference as well as the within-class coherence. While AA and FA consider rank average, we also included AM and FM, which use rank median. By using the median, we hoped to avoid any outlier effects. First, we simulated data and varied in the effect size and covariance structure of the signal genes. Effect size and covariance structure did not change the relative performance of each method, but overall, prediction accuracy was higher when signal genes were stronger and more correlated. Interestingly, TSP was consistently the worst feature selection method. This may be due to the classification scheme of the RF algorithm. RF favors nodes with high purity, which is indicated by low within-class variation. Conversely, TSP considers between-class differences, but does not directly compute within-class variation. Poor performance of TSP was also observed when we introduced a specific type of noise, consisting of genes with very low expression and a few normally-distributed outliers. Such genes simulate those that are naturally expressed at very low levels, such as tissue- or sex-specific genes. Fisher methods outperformed AM and AA, and TSP was the worst. This points to the importance of more feature information in building an accurate RF. Additionally, methods that used average rank were better than methods that used median rank, suggesting that the feature selection methods are not highly sensitive to outliers and skewed data.

After demonstrating the effectiveness of feature selection methods on simulated gene pairs, we then applied the methodology to real cancer data. First, we used the ranking system to combine breast tumor transcriptomic datasets. Previous large-scale meta-analyses have looked across 8 to 15 datasets with 1000–3000 patients [15–17], but we more than triple the sample size by combining 52 datasets for a total of over 10,000 samples. Gene pairs were used to validate known tumorigenic genes. When examining the top 100 gene pairs, *BSDC1*, *C3orf14*, *CDR2L*, *LRRC42*, *MEOX2*, *NRG2*, and *PLEKHA6*, and *SCARF1* were consistently identified by feature selection methods to be associated with cancer status. *C3orf14*, *CDR2L*, *LRRC42*, *MEOX2*, *NRG2*, and *SCARF1* have been previously associated with cancer. *C3orf14* has been shown to be differentially methylated in cervical and prostate cancer [18, 19] while *CDR2L* and *SCARF1* have been proposed to mediate immune response in ovarian and liver cancer, respectively [20–22]. *LRRC42* is a nuclear protein that is highly expressed in lung tumors and is indicative of heightened proliferation in vitro [23]. *LRRC42* is hypothesized to stabilize the methyl-CpG binding protein 1 (MeCP1) complex and regulate p21 transcription. Thus, it is not surprising that we observed high *LRRC42* in breast tumors. *MEOX2* is a transcription factor that has also been studied in cancer due to its growth arresting properties. *MEOX2* has been shown to inhibit cell migration via p21, induce apoptosis via BAX, and negatively regulate angiogenesis via nuclear factor kappa B (NF-κB) [24–27]. Our results support previous literature, as we observed a reduction in *MEOX2* expression in breast tumors. Finally, *NRG2* is a member of the neuregulin growth factor family that can bind HER2 to induce metastasis and modulate drug response [28–31]. Identification of known tumorigenic genes validates the ability of the feature selection methods to accurately filter gene pairs to return biologically relevant results.

In addition to confirming known carcinogenic genes, we also discovered new potential oncogenes. The role of *PLEKHA6* and *BSDC1* in carcinogenesis has not been fully uncovered. Evidence suggests that pleckstrin homology domain-containing proteins such as *PLEKHA6* are involved in intracellular signaling via G-protein-coupled receptors, protein kinase C, and phosphatidylinositol-3-kinase (PI3K) [32, 33]. Therefore, it is possible that aberrant *PLEKHA6* expression impacts cellular processes such as metabolism and apoptosis via PI3K activation or proliferation and differentiation by mitogen-activated protein kinase signaling. Similarly, little is known about the involvement of *BSDC1* in breast cancer. Initially discovered in 2002, BSD domains were first found in transcription factors and synaptic proteins [34]. The specific function of *BSDC1* remains unknown; however, copy number variations in *BSDC1* have been observed in ER-negative breast tumors [35], and sequence mutations in the gene have been associated with glioblastoma tumorigenesis [36]. We found that *BSDC1* was overexpressed in breast tumors, suggesting that *BSDC1* may be an important driver of carcinogenesis. Further experimentation should explicitly assess the biological function of *BSDC1* in order to validate its role as an oncogene and examine its potential as a molecular target in breast cancer.

We then divided patients by ER and HER2 status in order to validate the performance of feature selection methods in identifying relevant oncogenes as well as to test the ability of gene pairs to correctly stratify patients. First, we found that several genes were indicative of ER status, including *ESR1* itself as well as *ATP6AP2*, *CSNK1A1*, *INAVA*, *SOX11*, *TPM3*, and *U2AF1*. *SOX11* has been associated with ER status, as ER-negative tumors were shown to have lower levels of DNA methylation around the *SOX11* promoter was as well as higher gene expression [37]. Conversely, the tumorigenic roles of *ATP6AP2*, *CSNK1A1*, *INAVA*, *TPM3*, and *U2AF1* have been described, but it is unclear how they contribute to ER-positive and ER-negative phenotypes. *ATP6AP2* is a vacuolar proton pump that is commonly mutated in granular tumors, resulting in reduced acidification of endosomal compartments and acquisition of oncogenic traits [38]. *CSNK1A1* is a tumor suppressor gene that is involved in Wnt/β-catenin signaling [39]. Primarily studied in colon cancer, *INAVA* stabilizes adherens junctions and participates in inflammatory signaling [40, 41]. *TPM3* is a tropomyosin protein that binds actin filaments and stabilizes the cytoskeleton, and it has been shown to participate in epithelial-mesenchymal transition (EMT) [42]. Mutations in *U2AF1* in leukemia have been shown to impact splicing of hundreds of genes and have widespread consequences [43]. Future experimentation should focus on defining the roles of *ATP6AP2*, *CSNK1A1*, *INAVA*, *TPM3*, and *U2AF1* in ER signaling and understanding how the ER pathway interacts with general carcinogenic mechanisms.

For patient classification based on HER2 status, we found that HER2 was not identified by any feature selection method as a strong indicator of tumor status. HER2 status is often determined by protein levels measured by immunohistochemistry. Thus, our findings might suggest that HER2 protein levels are not strictly correlated with mRNA levels; however, this is not supported by a majority of the current literature [44–47]. Although HER2 alone may be a valid biomarker, other genes may have qualities that make them better suited for patient stratification using the feature selection techniques. The feature selection approaches favored genes with large between-class differences and within-class concordance. Furthermore, the methods were performed on gene pairs. Thus, selection as a top gene was highly dependent on the existence of a gene with inverse expression levels, such as a repressor or a protein involved in negative feedback. We hypothesize that the identified top genes fit these criteria. Specifically, *GRB7*, *PNMT*, *TCAP*, *TPM3*, and *U2AF1* gene expression were better indicators of HER2 status. Previous reports have identified co-amplification of *GRB7*, *PNMT*, and *TCAP* in HER2-positive breast cancer, specifically due to their location in the 17q12 region [13]. In particular, *GRB7* overexpression has been shown to potentiate HER2 amplification such that silencing the gene decreased proliferation and facilitated drug response to anti-HER2 drugs [48, 49]. On the other hand, *TPM3* and *U2AF1* have not been previously associated with HER2 status. It is interesting that these two genes were also identified as indicators of ER status. Previous literature has pointed to an inverse relationship between ER and HER2 status [50], so it is possible that *TPM3* and *U2AF1* represent a common mechanism that accounts for dysregulation in both systems.

After applying gene pairs to large-scale data integration, we next attempted to formulate a simple workflow for patient screening. In order to optimize treatment efficacy and patient prognosis, detection should preferably occur before the tumor becomes inoperable or the cancer has metastasized. We specifically examined bladder, ovarian, pancreatic, and prostate cancers because they are difficult to detect or have minimal screening procedures for asymptomatic individuals. Given their presence in biological fluids and relatively high stability, miRNA are promising biomarkers that have been previously explored in bladder [51, 52], ovarian [53], pancreatic [54], and prostate cancer [55]; however, few studies have presented circulating miRNA in a simplistic framework that can be readily translated to a clinical setting. We propose that miRNA pairs are ideal for clinical use. Unlike other biomarkers that use standardized units and defined cutoffs, miRNA pairs rely solely on relative within-patient values. This eliminates the need for specific measurement equipment, which is particularly convenient for gene expression. We first used feature selection to filter miRNA pairs then constructed full RF and BT models. However, our primary goal was to devise a transparent screening system, so we used salient rules from the full ensemble models to build STELs with clear, sequential decision rules. Simplifying the classifier slightly compromised accuracy, but for ovarian and pancreatic cancer, accuracy remained relatively high at 0.936 and 0.848, respectively. We provide preliminary evidence that miRNA pairs are suitable for cancer classification. Furthermore, STELs are not only highly interpretable, but they can also be updated to incorporate other demographic and clinical data to improve decision rules and classification accuracy.

Gene pairs have strengths and limitations that should be addressed in future investigation. As opposed to traditional methodology, the use of gene pairs in clinical practice has the potential to eliminate the need for standardized measurement techniques, validated cutoffs, and data normalization. Researchers can also utilize gene pairs and rank-based methodology to integrate datasets across platforms and experimental cohorts. One major weakness of gene pairs is the huge expansion of the feature space and the corresponding increase in computational time and susceptibility to overfitting. Genomic data already suffers from high dimensionality, where features vastly outnumber observations (*p* >  > *n*). Gene pairs amplify this problem. We use feature selection strategies that exhaustively calculate scores for every gene pair, but other approaches might reduce computational time either by eliminating single genes before pairs are queried or by sampling gene pairs in a non-exhaustive manner. Alternatively, future work might focus on parallelizing calculations to increase computational efficiency. Although there are still several avenues by which to improve and apply gene pair analysis, we provide foundational insight into the performance of feature selection methods on gene pair data and demonstrate two powerful applications.

## Conclusion

In the current study, we examined feature selection approaches and novel biological and clinical applications for gene pairs. We first found that classification using gene pairs benefited from retaining maximal information during feature selection. Incorporating the magnitude of gene expression change as well as the within-class variance using Fisher scores improved classification accuracy, especially when data contained many genes with low expression. We then demonstrated the effectiveness of using gene ranking by combining breast cancer datasets to create a large cohort of 10,350 tumors. Gene pairs were then used to discover new oncogenes related to both carcinogenesis and ER/HER2 status, including *BSDC1* and *U2AF1*. Lastly, we constructed STELs using circulating miRNA pairs in order to provide a transparent framework for pancreatic and ovarian cancer screening. Our approach is highly adaptable for clinical use because it is invariant to measurement platform, normalization, and specific cutoff values. Collectively, we show that gene pairs can overcome the limitations presented by genomic data and accurately classify tumors in an interpretable manner.

## Methods

### Feature selection methods

The goal of feature selection for gene pairs was to reduce the feature space to avoid overfitting and increase computational efficiency. We used five filter methods that retained different amounts of information in their score calculation. For each approach, we can consider a cohort of *N* samples, each having set of *p* genes whose expression is measured in a transcriptomic profile *X*_*n*_ = {*x*_1_, *x*_2_, …, *x*_*p*_}. For all approaches, within-sample pairwise comparisons are made, such that the within-sample rankings are sufficient to perform all calculations. The ranks of {*x*_1_, *x*_2_, …, *x*_*p*_} can be denoted as {*r*_1_, *r*_2_, …, *r*_*p*_}. Additionally, each *X* may belong to a particular class *C* = {*c*_1_, *c*_2_}. We only consider binary classification in this study, but these methods can be extended to multi-class problems.

### Top-scoring pairs (TSP)

The TSP algorithm makes within-sample pairwise comparisons, calculates the probability that rank is higher in one gene or the another, and compares probabilities between classes [8]. For the gene pair (*x*_*i*_, *x*_*j*_) having within-samples ranks (*r*_*i*_, *r*_*j*_), the calculation of the TSP score proceeds by first observing the probability of *r*_*i*_ < *r*_*j*_ in class 1: *p*_*ij*_(1) = *P*(*r*_*i*_ < *r*_*j*_| *c*_1_)*. The same calculation is performed for the prob*ability of *r*_*i*_ < *r*_*j*_ in class 2: *p*_*ij*_(2) = *P*(*r*_*i*_ < *r*_*j*_| *c*_2_). The difference between the two probabilities can then be calculated to get the pair score:

$$ {TSP}_{ij}=\left|{p}_{ij}(1)-{p}_{ij}(2)\right|. $$

### Absolute average (AA) and absolute median (AM)

TSP is quite simplistic in that it calculates probabilities but does not consider any other information about the nature of the within-sample gene expression differences. Instead, we can consider the magnitude of the rank difference between two genes. This can be achieved by first calculating the within-sample pairwise differences between gene ranks. For (*r*_*i*_, *r*_*j*_) the rank difference can be calculated as *R*_*ij*_ = *r*_*i*_ − *r*_*j*_. This is repeated for all samples. Then we can call *R*_*ij*_(1) the set of rank differences for samples in class 1 samples, and *R*_*ij*_(2) the set of rank differences for the samples in class 2. The AA score can be computed by taking the average rank difference for each class and then calculating the absolute difference:

$$ {AA}_{ij}=\left|\overline{R_{ij}(1)}-\overline{R_{ij}(2)}\right|. $$

The AM score can be computed by taking the absolute difference between the class medians:

$$ {AM}_{ij}=\left| median\left[{R}_{ij}(1)\right]- median\left[{R}_{ij}(2)\right]\right|. $$

By using the median, we attempt to minimize the effect of outliers.

### Fisher average (FA) and fisher median (FM)

In addition to considering the magnitude of the rank change, we can also incorporate the within-class variance. This can be achieved via calculation of Fisher scores, which favor features with large distances between data points from different classes and small distances between data points from the same class [56]. We calculate a classic Fisher score using average ranks:

$$ {FA}_{ij}=\frac{n_1\left(\overline{R_{ij}(1)}-\overline{R_{ij}}\right)+{n}_2\left(\overline{R_{ij}(2)}-\overline{R_{ij}}\right)}{n_1{\sigma}_{ij}^2(1)+{n}_2{\sigma}_{ij}^2(2)}. $$

We also account for outliers and skewed data by using median ranks:

$$ {FM}_{ij}=\frac{n_1\left( median\left[{R}_{ij}(1)\right]- median\left[{R}_{ij}\right]\right)+{n}_2\left( median\left[{R}_{ij}(2)\right]- median\left[{R}_{ij}\right]\right)}{n_1{\sigma}_{ij}^2(1)+{n}_2{\sigma}_{ij}^2(2)}. $$

In both equations, *R*_*ij*_ is the average rank difference for all samples in both classes. *n*_1_ and *n*_2_ represent the number of samples in class 1 and class 2, respectively. ${\sigma}_{ij}^2(1)$ and ${\sigma}_{ij}^2(2)$ represent the variance in rank differences in class 1 and class 2, respectively.

#### Classifiers

After feature selection, classification was performed using ensemble classifiers. Although gene ranks were used to choose features, we transformed values into binary categorical variables before classification. Each feature, *f*_*ij*_, represented a gene pair. When *r*_*i*_ < *r*_*j*_, a value of 1 was assigned, and when *r*_*i*_ < *r*_*j*_ a value of − 1 was assigned. This transformation was performed in order to minimize data processing and broadly represent measurements that could be taken on a variety of platforms. Instead of requiring the exact expression values or rankings across a large spectrum of genes, using a binary variable only requires knowledge of the relative expression between two genes. We proposed that this would be particularly beneficial for use in a clinical setting.

### Random forest (RF)

Random forest is an ensemble method that fits a collection of de-correlated decision trees on bootstrapped training samples and then tallies votes over the entire forest to get a prediction for each observation [57]. We tuned our models by using the ‘caret’ package in R. Specifically, at each split in a tree, we considered *m* predictors (*mtry*), for which we tried values ranging from 1 to 10. The best fit was chosen based on out-of-bag error and was used to classify test data.

### Boosted tree (BT)

Gradient boosting improves robustness of a decision tree by sequentially growing trees based on the residuals of the previous tree [58]. We tuned three model parameters, including the total number of trees, the shrinkage parameter, and the number of splits (depth) in each tree. The number of trees took values of 100, 500, or 1000, shrinkage was set at 0.01 or 0.1, and the number of splits ranged from 1 to 5. The model with the best accuracy using was chosen using 5-fold cross validation was used to predict on the testing data.

### Simplified tree ensemble learner (STEL)

Finally, we attempted to reduce ensemble classifiers into simplified tree ensemble learners (STELs). Although ensemble methods are robust and provide better classification accuracy than single decision trees, they are not highly interpretable because they average over a collection of trees. On the other hand, the structure of single decision trees is easy to explain, can be presented in graphical format, and mirrors stepwise processes used for clinical decision-making. Thus, we built full ensemble learners, extracted non-redundant decision rules, and compiled them into STELs.

**Fig. 8:**
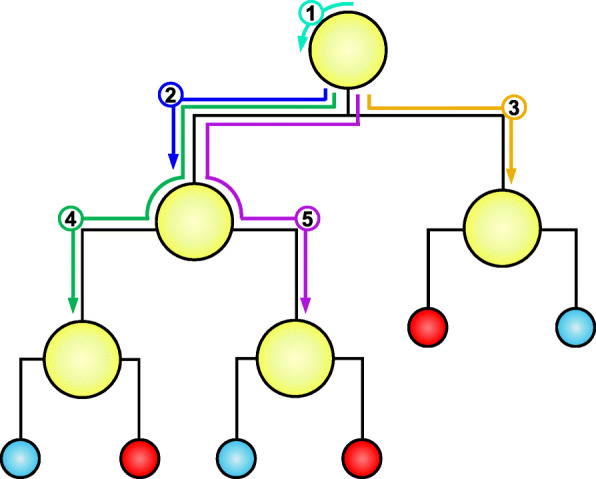
Rule extraction from a decision tree. Starting at the root of a tree, the first decision rule is extracted (labeled ‘1’). The algorithm moves one level deeper in the tree and combines the first rule with each of the nodes at the current level to create new extracted rules. These steps are repeated until a leaf is reached and all paths from root to leaves have been traversed. Individual decision rules are denoted as yellow nodes. Leaves are blue and red circles, denoting a binary outcome. Each extracted rule is numbered and consists of the combination of rules along its path

In order to extract rules, the R package ‘inTrees’ was used [59]. The extracted rules are combinations of rules from each decision tree of the ensemble. Starting at the root of a tree, the first decision rule is extracted. The algorithm moves to the second rule and combines it with the first rule to create a new rule. These steps are repeated until a leaf is reached. The process is then repeated until all paths from root to leaves have been traversed for every tree of the ensemble (Fig. 8).

Rules are then prioritized based on their frequency, error, and length. Frequency refers to the proportion of cases that satisfy a rule. Error indicates the classification error by the single rule. Length refers to the number of features included in the rule. Rules with high frequency and low error and length are preferable to avoid overfitting, increase accuracy, and facilitate interpretability. Thus, rules with frequency less than 0.01 and length greater than 6 were not considered.

Finally, STELs can be built by iteratively adding extracted rules to a tree. The set of rules is first evaluated to find the one with the minimum error. Ties are broken using frequency and then using length. Observations satisfying the current rule are removed and the next rule is chosen using the remaining data. The algorithm stops when all remaining rules return an error that is greater than the error produced by simply choosing the most frequent class. The output is an ordered rule list that can be easily depicted as a series of well-defined steps and resulting outcomes.

## Supplementary information

**Additional file 1.** Top gene pairs (breast cancer vs control). This file contains raw calculated data of the top 100 gene pairs and feature selection scores for the breast cancer and control comparison.

**Additional file 2.** Top gene pairs (ER status). This file contains raw calculated data of the top 100 gene pairs and feature selection scores for the ER-positive and ER-negative comparison.

**Additional file 3.** Top gene pairs (HER2 status). This file contains raw calculated data of the top 100 gene pairs and feature selection scores for the HER2-positive and HER2-negative comparison.

## Data Availability

The datasets analyzed in the current study are available in the Gene Expression Omnibus (GEO) repository, https://www.ncbi.nlm.nih.gov/geo/. All accession numbers to all datasets are included in this published article.

## Abbreviations

AA: Absolute average
AM: Absolute median
*ARHGEF15*: Rho guanine nucleotide exchange factor 15
*ATP6AP2*: ATPase H+ transporting accessory protein 2
*ATP6V1B2*: ATPase H+ transporting V1 subunit B2
*BSDC1*: BSD domain containing 1
BT: Boosted tree
*C3orf14*: Chromosome 3 open reading frame 14
*CDR2L*: Cerebellar degeneration related protein 2 like
*CSAD*: Cysteine sulfinic acid decarboxylase
*CSNK1A1*: Casein kinase 1 alpha 1
*CUEDC1*: CUE domain containing C1
*CYP26B1*: Cytochrome P450 family 26 subfamily B1
*DMD*: Dystrophin
EMT: Epithelial-mesenchymal transition
*ESR1*: Estrogen receptor 1
FA: Fisher average
*FAT4*: FAT atypical cadherin 4
FM: Fisher median
HER2: human epidermal growth factor receptor 2
*INAVA*: Innate immunity activator
*LRRC42*: Leucine rich repeat containing 42
MeCP1: Methyl-CpG binding protein 1
*MEOX1*: Mesenchyme homeobox 1
*MEOX2*: Mesenchyme homeobox 2
miRNA: microrna
NF-κB: Nuclear factor kappa B
*NR3C2*: Nuclear receptor subfamily 3 group C2
*NRG2*: Neuregulin 2
PI3K: Phosphatidylinositol-3-kinase
*PLEKHA6*: Plekstrin homology domain containing A6
*PNMT*: Phenylethanolamine N-methyltransferase
*POU6F1*: POU class 6 homeobox 1
RF: Random forest
*SCARF1*: Scavenger receptor class F1
*SEMA3G*: Semaphoring 3G
*SOCS5*: Suppressor of cytokine signaling 5
*SOX11*: SRY-box 11
STEL: Simplified tree ensemble learner
SVM: Support vector machine
*TCAP*: Titin-cap
*TPM3*: Tropomyosin 3
TSP: Top scoring pairs
*U2AF1*: U2 small nuclear RNA auxiliary factor
